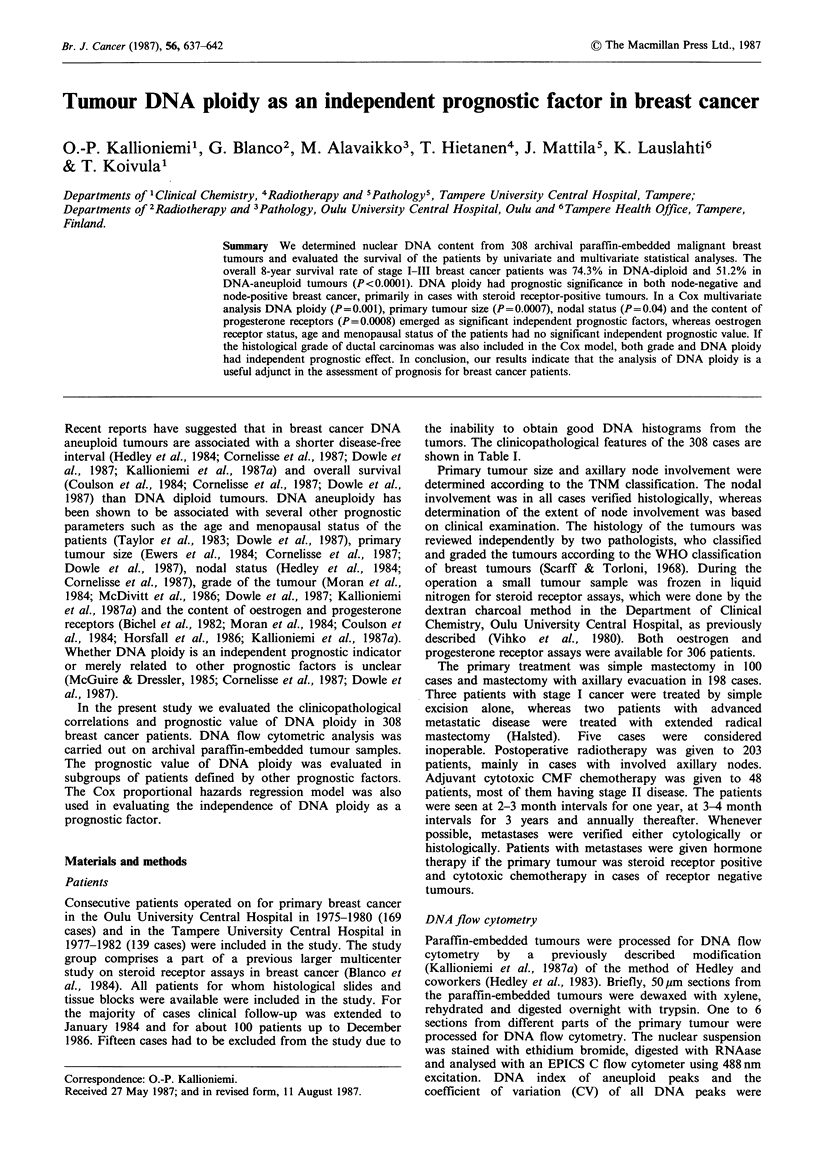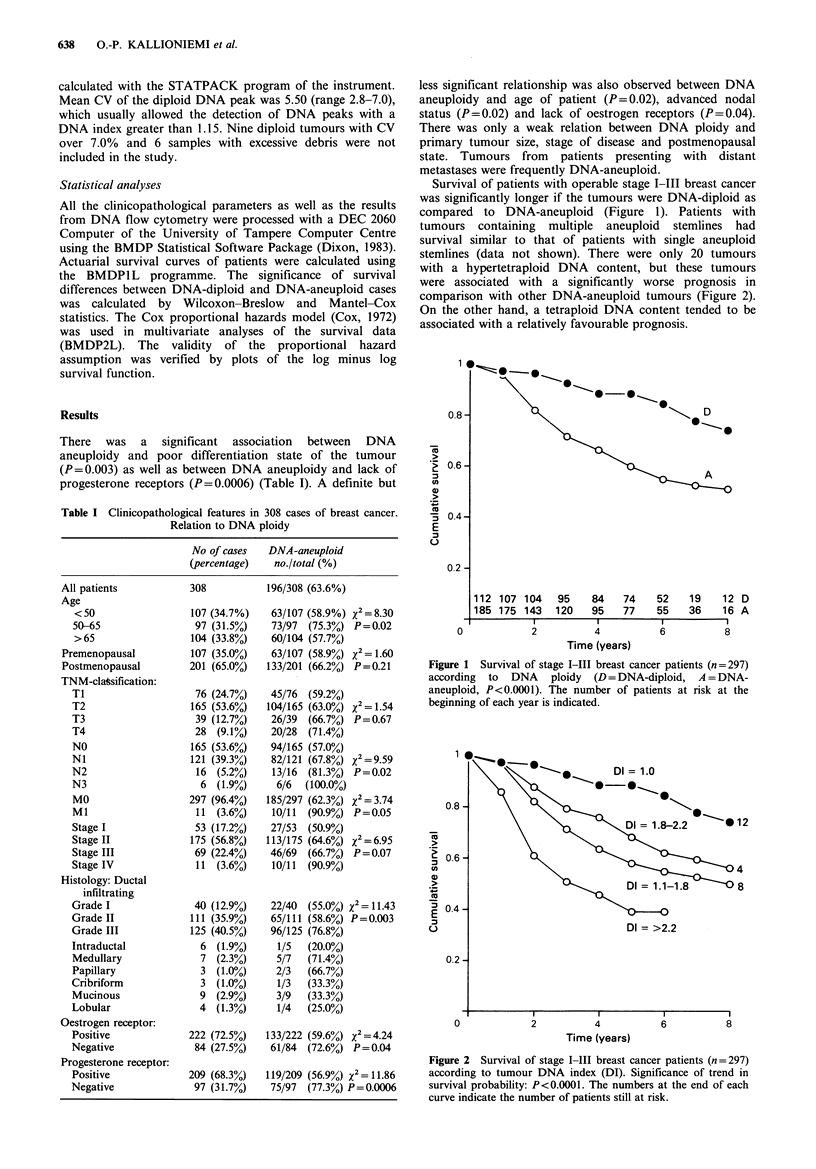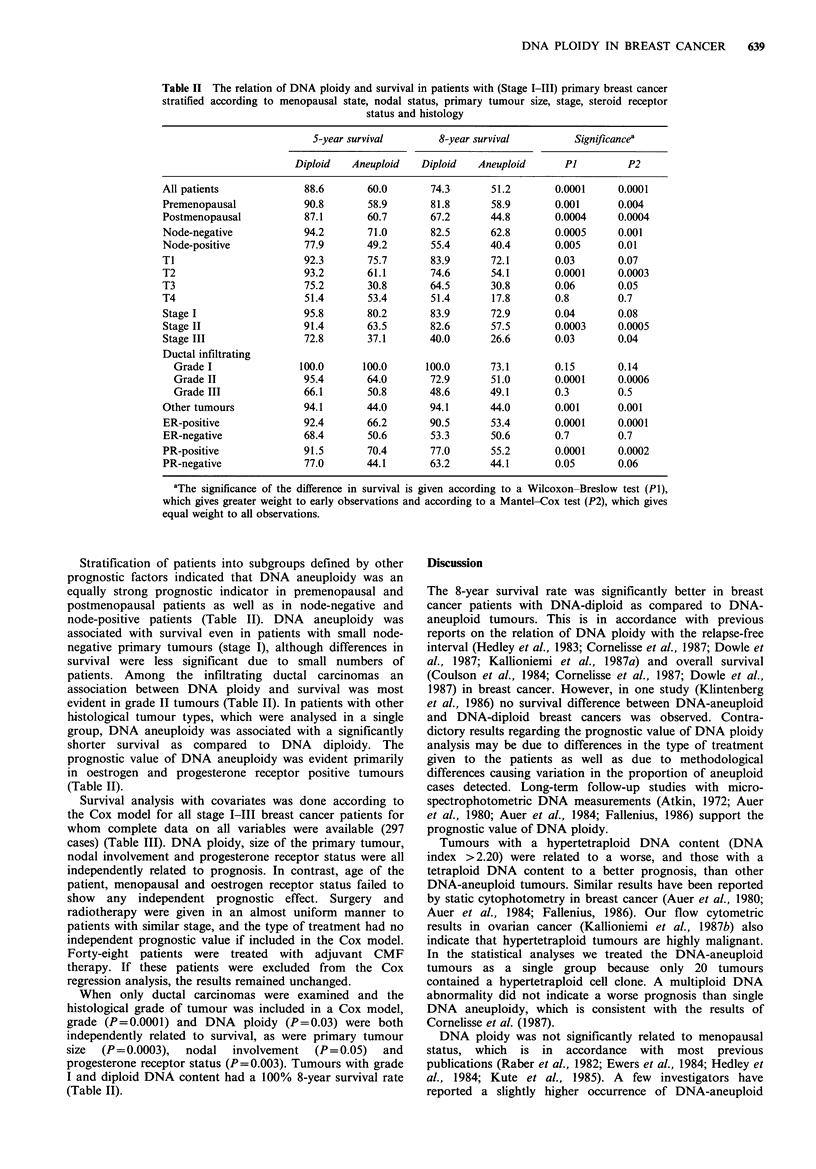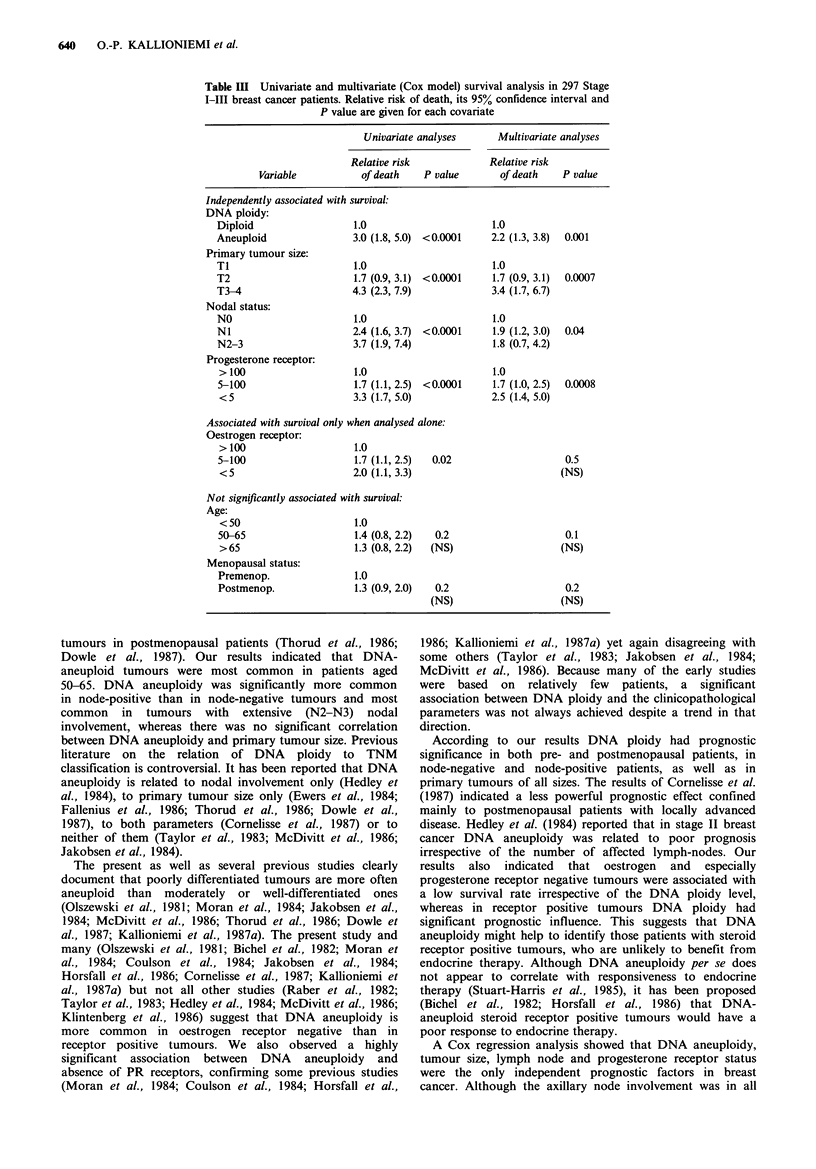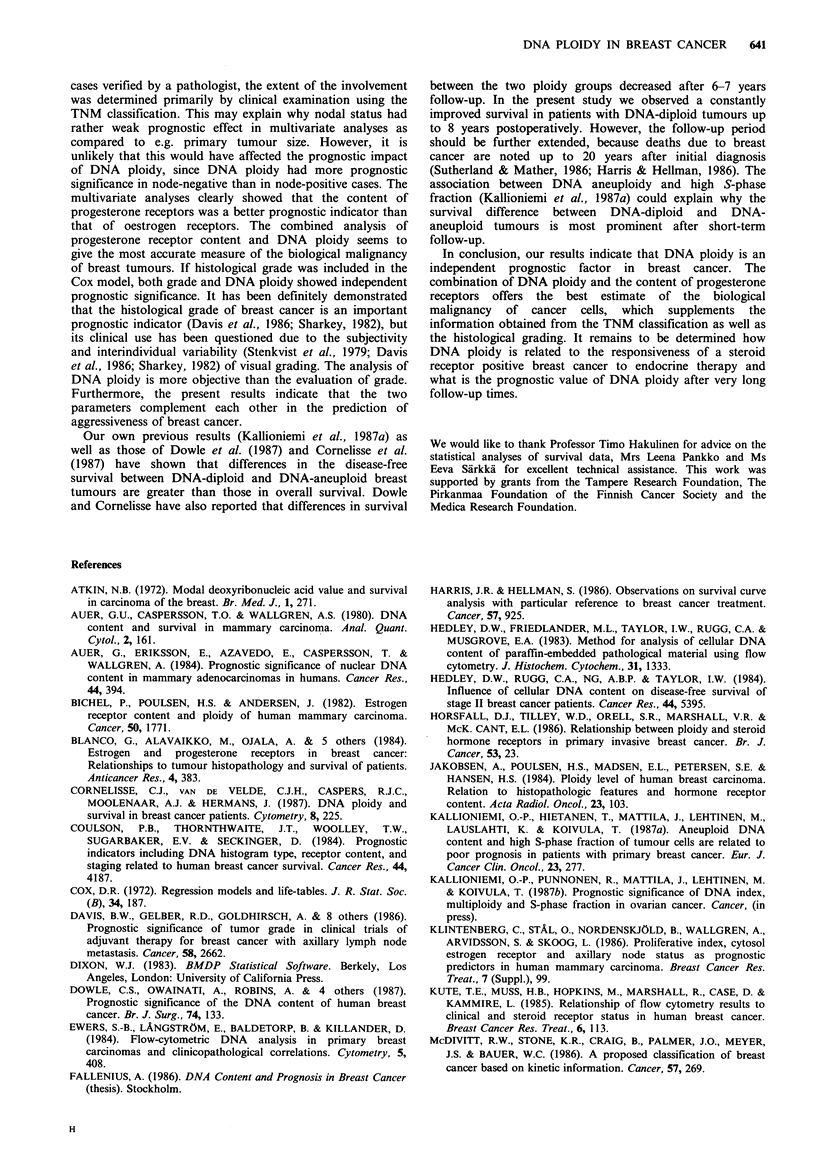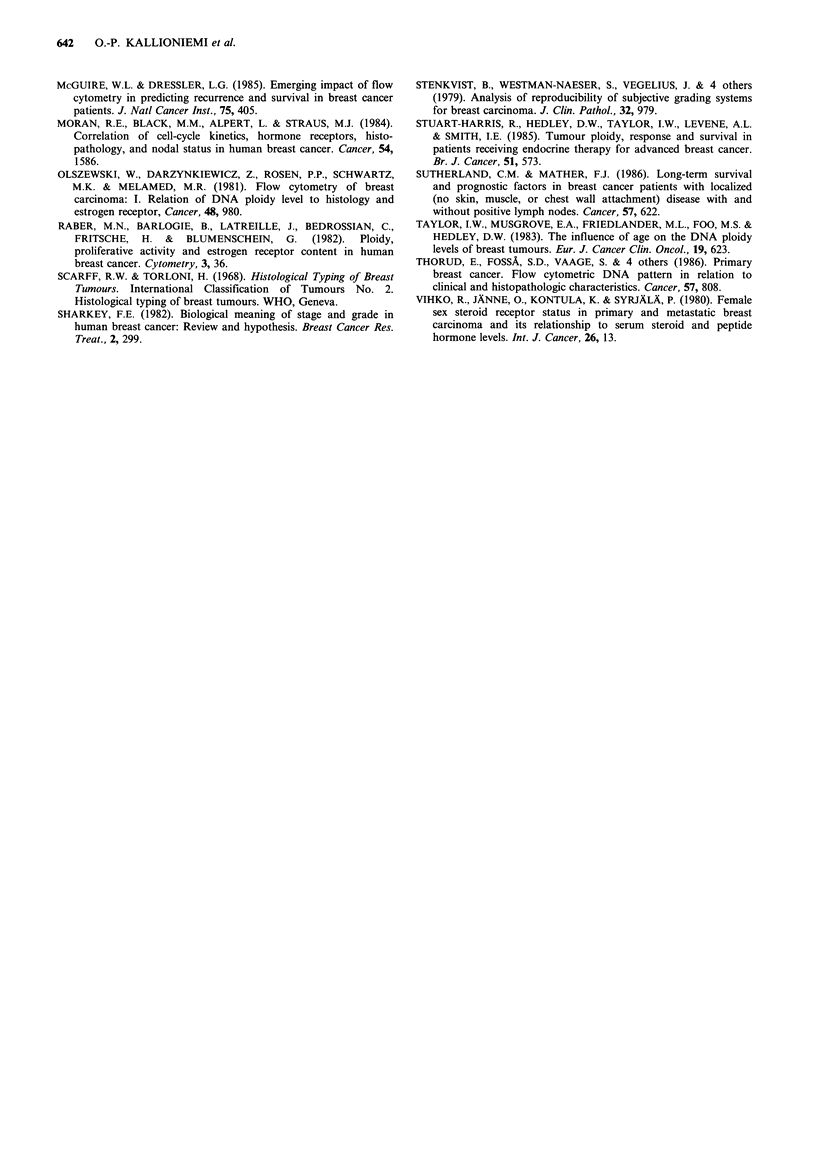# Tumour DNA ploidy as an independent prognostic factor in breast cancer.

**DOI:** 10.1038/bjc.1987.258

**Published:** 1987-11

**Authors:** O. P. Kallioniemi, G. Blanco, M. Alavaikko, T. Hietanen, J. Mattila, K. Lauslahti, T. Koivula

**Affiliations:** Department of Clinical Chemistry, Tampere University Central Hospital, Finland.

## Abstract

We determined nuclear DNA content from 308 archival paraffin-embedded malignant breast tumours and evaluated the survival of the patients by univariate and multivariate statistical analyses. The overall 8-year survival rate of stage I-III breast cancer patients was 74.3% in DNA-diploid and 51.2% in DNA-aneuploid tumours (P less than 0.0001). DNA ploidy had prognostic significance in both node-negative and node-positive breast cancer, primarily in cases with steroid receptor-positive tumours. In a Cox multivariate analysis DNA ploidy (P = 0.001), primary tumour size (P = 0.0007), nodal status (P = 0.04) and the content of progesterone receptors (P = 0.0008) emerged as significant independent prognostic factors, whereas oestrogen receptor status, age and menopausal status of the patients had no significant independent prognostic value. If the histological grade of ductal carcinomas was also included in the Cox model, both grade and DNA ploidy had independent prognostic effect. In conclusion, our results indicate that the analysis of DNA ploidy is a useful adjunct in the assessment of prognosis for breast cancer patients.


*Br. J. Cancer* (1987), **56**, 637–642 © The Macmillan Press Ltd., 1987

### Tumour DNA ploidy as an independent prognostic factor in breast cancer

O.-P. Kallioniemi[1], G. Blanco[2], M. Alavaikko[3], T. Hietanen[4], J. Mattila[5], K. Lauslahti[6] & T. Koivula[1]

*Departments of [1]Clinical Chemistry, [4]Radiotherapy and [5]Pathology[5], Tampere University Central Hospital, Tampere; Departments of [2]Radiotherapy and [3]Pathology, Oulu University Central Hospital, Oulu and [6]Tampere Health Office, Tampere, Finland.*

**Summary** We determined nuclear DNA content from 308 archival paraffin-embedded malignant breast tumours and evaluated the survival of the patients by univariate and multivariate statistical analyses. The overall 8-year survival rate of stage I–III breast cancer patients was 74.3% in DNA-diploid and 51.2% in DNA-aneuploid tumours ($P<0.0001$). DNA ploidy had prognostic significance in both node-negative and node-positive breast cancer, primarily in cases with steroid receptor-positive tumours. In a Cox multivariate analysis DNA ploidy ($P=0.001$), primary tumour size ($P=0.0007$), nodal status ($P=0.04$) and the content of progesterone receptors ($P=0.0008$) emerged as significant independent prognostic factors, whereas oestrogen receptor status, age and menopausal status of the patients had no significant independent prognostic value. If the histological grade of ductal carcinomas was also included in the Cox model, both grade and DNA ploidy had independent prognostic effect. In conclusion, our results indicate that the analysis of DNA ploidy is a useful adjunct in the assessment of prognosis for breast cancer patients.

Recent reports have suggested that in breast cancer DNA aneuploid tumours are associated with a shorter disease-free interval (Hedley *et al.*, 1984; Cornelisse *et al.*, 1987; Dowle *et al.*, 1987; Kallioniemi *et al.*, 1987*a*) and overall survival (Coulson *et al.*, 1984; Cornelisse *et al.*, 1987; Dowle *et al.*, 1987) than DNA diploid tumours. DNA aneuploidy has been shown to be associated with several other prognostic parameters such as the age and menopausal status of the patients (Taylor *et al.*, 1983; Dowle *et al.*, 1987), primary tumour size (Ewers *et al.*, 1984; Cornelisse *et al.*, 1987; Dowle *et al.*, 1987), nodal status (Hedley *et al.*, 1984; Cornelisse *et al.*, 1987), grade of the tumour (Moran *et al.*, 1984; McDivitt *et al.*, 1986; Dowle *et al.*, 1987; Kallioniemi *et al.*, 1987*a*) and the content of oestrogen and progesterone receptors (Bichel *et al.*, 1982; Moran *et al.*, 1984; Coulson *et al.*, 1984; Horsfall *et al.*, 1986; Kallioniemi *et al.*, 1987*a*). Whether DNA ploidy is an independent prognostic indicator or merely related to other prognostic factors is unclear (McGuire & Dressler, 1985; Cornelisse *et al.*, 1987; Dowle *et al.*, 1987).

In the present study we evaluated the clinicopathological correlations and prognostic value of DNA ploidy in 308 breast cancer patients. DNA flow cytometric analysis was carried out on archival paraffin-embedded tumour samples. The prognostic value of DNA ploidy was evaluated in subgroups of patients defined by other prognostic factors. The Cox proportional hazards regression model was also used in evaluating the independence of DNA ploidy as a prognostic factor.

### Materials and methods

### *Patients*

Consecutive patients operated on for primary breast cancer in the Oulu University Central Hospital in 1975–1980 (169 cases) and in the Tampere University Central Hospital in 1977–1982 (139 cases) were included in the study. The study group comprises a part of a previous larger multicenter study on steroid receptor assays in breast cancer (Blanco *et al.*, 1984). All patients for whom histological slides and tissue blocks were available were included in the study. For the majority of cases clinical follow-up was extended to January 1984 and for about 100 patients up to December 1986. Fifteen cases had to be excluded from the study due to the inability to obtain good DNA histograms from the tumors. The clinicopathological features of the 308 cases are shown in Table I.

Primary tumour size and axillary node involvement were determined according to the TNM classification. The nodal involvement was in all cases verified histologically, whereas determination of the extent of node involvement was based on clinical examination. The histology of the tumours was reviewed independently by two pathologists, who classified and graded the tumours according to the WHO classification of breast tumours (Scarff & Torloni, 1968). During the operation a small tumour sample was frozen in liquid nitrogen for steroid receptor assays, which were done by the dextran charcoal method in the Department of Clinical Chemistry, Oulu University Central Hospital, as previously described (Vihko *et al.*, 1980). Both oestrogen and progesterone receptor assays were available for 306 patients.

The primary treatment was simple mastectomy in 100 cases and mastectomy with axillary evacuation in 198 cases. Three patients with stage I cancer were treated by simple excision alone, whereas two patients with advanced metastatic disease were treated with extended radical mastectomy (Halsted). Five cases were considered inoperable. Postoperative radiotherapy was given to 203 patients, mainly in cases with involved axillary nodes. Adjuvant cytotoxic CMF chemotherapy was given to 48 patients, most of them having stage II disease. The patients were seen at 2–3 month intervals for one year, at 3–4 month intervals for 3 years and annually thereafter. Whenever possible, metastases were verified either cytologically or histologically. Patients with metastases were given hormone therapy if the primary tumour was steroid receptor positive and cytotoxic chemotherapy in cases of receptor negative tumours.

### *DNA flow cytometry*

Paraffin-embedded tumours were processed for DNA flow cytometry by a previously described modification (Kallioniemi *et al.*, 1987*a*) of the method of Hedley and coworkers (Hedley *et al.*, 1983). Briefly, 50 $\mu$m sections from the paraffin-embedded tumours were dewaxed with xylene, rehydrated and digested overnight with trypsin. One to 6 sections from different parts of the primary tumour were processed for DNA flow cytometry. The nuclear suspension was stained with ethidium bromide, digested with RNAase and analysed with an EPICS C flow cytometer using 488 nm excitation. DNA index of aneuploid peaks and the coefficient of variation (CV) of all DNA peaks were

Correspondence: O.-P. Kallioniemi.
Received 27 May 1987; and in revised form, 11 August 1987.

calculated with the STATPACK program of the instrument. Mean CV of the diploid DNA peak was 5.50 (range 2.8–7.0), which usually allowed the detection of DNA peaks with a DNA index greater than 1.15. Nine diploid tumours with CV over 7.0% and 6 samples with excessive debris were not included in the study.

### *Statistical analyses*

All the clinicopathological parameters as well as the results from DNA flow cytometry were processed with a DEC 2060 Computer of the University of Tampere Computer Centre using the BMDP Statistical Software Package (Dixon, 1983). Actuarial survival curves of patients were calculated using the BMDP1L programme. The significance of survival differences between DNA-diploid and DNA-aneuploid cases was calculated by Wilcoxon–Breslow and Mantel–Cox statistics. The Cox proportional hazards model (Cox, 1972) was used in multivariate analyses of the survival data (BMDP2L). The validity of the proportional hazard assumption was verified by plots of the log minus log survival function.

### Results

There was a significant association between DNA aneuploidy and poor differentiation state of the tumour ($P=0.003$) as well as between DNA aneuploidy and lack of progesterone receptors ($P=0.0006$) (Table I). A definite but less significant relationship was also observed between DNA aneuploidy and age of patient ($P=0.02$), advanced nodal status ($P=0.02$) and lack of oestrogen receptors ($P=0.04$). There was only a weak relation between DNA ploidy and primary tumour size, stage of disease and postmenopausal state. Tumours from patients presenting with distant metastases were frequently DNA-aneuploid.

Survival of patients with operable stage I–III breast cancer was significantly longer if the tumours were DNA-diploid as compared to DNA-aneuploid (Figure 1). Patients with tumours containing multiple aneuploid stemlines had survival similar to that of patients with single aneuploid stemlines (data not shown). There were only 20 tumours with a hypertetraploid DNA content, but these tumours were associated with a significantly worse prognosis in comparison with other DNA-aneuploid tumours (Figure 2). On the other hand, a tetraploid DNA content tended to be associated with a relatively favourable prognosis.

**Table I** Clinicopathological features in 308 cases of breast cancer. Relation to DNA ploidy

| | *No of cases (percentage)* | *DNA-aneuploid no./total* (%) | |
|---|---|---|---|
| All patients | 308 | 196/308 (63.6%) | |
| Age | | | |
| <50 | 107 (34.7%) | 63/107 (58.9%) | $\chi^2=8.30$ |
| 50–65 | 97 (31.5%) | 73/97 (75.3%) | $P=0.02$ |
| >65 | 104 (33.8%) | 60/104 (57.7%) | |
| Premenopausal | 107 (35.0%) | 63/107 (58.9%) | $\chi^2=1.60$ |
| Postmenopausal | 201 (65.0%) | 133/201 (66.2%) | $P=0.21$ |
| TNM-classification: | | | |
| T1 | 76 (24.7%) | 45/76 (59.2%) | |
| T2 | 165 (53.6%) | 104/165 (63.0%) | $\chi^2=1.54$ |
| T3 | 39 (12.7%) | 26/39 (66.7%) | $P=0.67$ |
| T4 | 28 (9.1%) | 20/28 (71.4%) | |
| N0 | 165 (53.6%) | 94/165 (57.0%) | |
| N1 | 121 (39.3%) | 82/121 (67.8%) | $\chi^2=9.59$ |
| N2 | 16 (5.2%) | 13/16 (81.3%) | $P=0.02$ |
| N3 | 6 (1.9%) | 6/6 (100.0%) | |
| M0 | 297 (96.4%) | 185/297 (62.3%) | $\chi^2=3.74$ |
| M1 | 11 (3.6%) | 10/11 (90.9%) | $P=0.05$ |
| Stage I | 53 (17.2%) | 27/53 (50.9%) | |
| Stage II | 175 (56.8%) | 113/175 (64.6%) | $\chi^2=6.95$ |
| Stage III | 69 (22.4%) | 46/69 (66.7%) | $P=0.07$ |
| Stage IV | 11 (3.6%) | 10/11 (90.9%) | |
| Histology: Ductal infiltrating | | | |
| Grade I | 40 (12.9%) | 22/40 (55.0%) | $\chi^2=11.43$ |
| Grade II | 111 (35.9%) | 65/111 (58.6%) | $P=0.003$ |
| Grade III | 125 (40.5%) | 96/125 (76.8%) | |
| Intraductal | 6 (1.9%) | 1/5 (20.0%) | |
| Medullary | 7 (2.3%) | 5/7 (71.4%) | |
| Papillary | 3 (1.0%) | 2/3 (66.7%) | |
| Cribriform | 3 (1.0%) | 1/3 (33.3%) | |
| Mucinous | 9 (2.9%) | 3/9 (33.3%) | |
| Lobular | 4 (1.3%) | 1/4 (25.0%) | |
| Oestrogen receptor: | | | |
| Positive | 222 (72.5%) | 133/222 (59.6%) | $\chi^2=4.24$ |
| Negative | 84 (27.5%) | 61/84 (72.6%) | $P=0.04$ |
| Progesterone receptor: | | | |
| Positive | 209 (68.3%) | 119/209 (56.9%) | $\chi^2=11.86$ |
| Negative | 97 (31.7%) | 75/97 (77.3%) | $P=0.0006$ |

**Figure 1** Survival of stage I–III breast cancer patients ($n=297$) according to DNA ploidy ($D$=DNA-diploid, $A$=DNA-aneuploid, $P<0.0001$). The number of patients at risk at the beginning of each year is indicated.

**Figure 2** Survival of stage I–III breast cancer patients ($n=297$) according to tumour DNA index (DI). Significance of trend in survival probability: $P<0.0001$. The numbers at the end of each curve indicate the number of patients still at risk.

**Table II** The relation of DNA ploidy and survival in patients with (Stage I–III) primary breast cancer stratified according to menopausal state, nodal status, primary tumour size, stage, steroid receptor status and histology

| | *5-year survival* | | *8-year survival* | | *Significance*[a] | |
|---|---|---|---|---|---|---|
| | *Diploid* | *Aneuploid* | *Diploid* | *Aneuploid* | *P1* | *P2* |
| All patients | 88.6 | 60.0 | 74.3 | 51.2 | 0.0001 | 0.0001 |
| Premenopausal | 90.8 | 58.9 | 81.8 | 58.9 | 0.001 | 0.004 |
| Postmenopausal | 87.1 | 60.7 | 67.2 | 44.8 | 0.0004 | 0.0004 |
| Node-negative | 94.2 | 71.0 | 82.5 | 62.8 | 0.0005 | 0.001 |
| Node-positive | 77.9 | 49.2 | 55.4 | 40.4 | 0.005 | 0.01 |
| T1 | 92.3 | 75.7 | 83.9 | 72.1 | 0.03 | 0.07 |
| T2 | 93.2 | 61.1 | 74.6 | 54.1 | 0.0001 | 0.0003 |
| T3 | 75.2 | 30.8 | 64.5 | 30.8 | 0.06 | 0.05 |
| T4 | 51.4 | 53.4 | 51.4 | 17.8 | 0.8 | 0.7 |
| Stage I | 95.8 | 80.2 | 83.9 | 72.9 | 0.04 | 0.08 |
| Stage II | 91.4 | 63.5 | 82.6 | 57.5 | 0.0003 | 0.0005 |
| Stage III | 72.8 | 37.1 | 40.0 | 26.6 | 0.03 | 0.04 |
| Ductal infiltrating | | | | | | |
| Grade I | 100.0 | 100.0 | 100.0 | 73.1 | 0.15 | 0.14 |
| Grade II | 95.4 | 64.0 | 72.9 | 51.0 | 0.0001 | 0.0006 |
| Grade III | 66.1 | 50.8 | 48.6 | 49.1 | 0.3 | 0.5 |
| Other tumours | 94.1 | 44.0 | 94.1 | 44.0 | 0.001 | 0.001 |
| ER-positive | 92.4 | 66.2 | 90.5 | 53.4 | 0.0001 | 0.0001 |
| ER-negative | 68.4 | 50.6 | 53.3 | 50.6 | 0.7 | 0.7 |
| PR-positive | 91.5 | 70.4 | 77.0 | 55.2 | 0.0001 | 0.0002 |
| PR-negative | 77.0 | 44.1 | 63.2 | 44.1 | 0.05 | 0.06 |

[a]The significance of the difference in survival is given according to a Wilcoxon–Breslow test (*P*1), which gives greater weight to early observations and according to a Mantel–Cox test (*P*2), which gives equal weight to all observations.

Stratification of patients into subgroups defined by other prognostic factors indicated that DNA aneuploidy was an equally strong prognostic indicator in premenopausal and postmenopausal patients as well as in node-negative and node-positive patients (Table II). DNA aneuploidy was associated with survival even in patients with small node-negative primary tumours (stage I), although differences in survival were less significant due to small numbers of patients. Among the infiltrating ductal carcinomas an association between DNA ploidy and survival was most evident in grade II tumours (Table II). In patients with other histological tumour types, which were analysed in a single group, DNA aneuploidy was associated with a significantly shorter survival as compared to DNA diploidy. The prognostic value of DNA aneuploidy was evident primarily in oestrogen and progesterone receptor positive tumours (Table II).

Survival analysis with covariates was done according to the Cox model for all stage I–III breast cancer patients for whom complete data on all variables were available (297 cases) (Table III). DNA ploidy, size of the primary tumour, nodal involvement and progesterone receptor status were all independently related to prognosis. In contrast, age of the patient, menopausal and oestrogen receptor status failed to show any independent prognostic effect. Surgery and radiotherapy were given in an almost uniform manner to patients with similar stage, and the type of treatment had no independent prognostic value if included in the Cox model. Forty-eight patients were treated with adjuvant CMF therapy. If these patients were excluded from the Cox regression analysis, the results remained unchanged.

When only ductal carcinomas were examined and the histological grade of tumour was included in a Cox model, grade ($P=0.0001$) and DNA ploidy ($P=0.03$) were both independently related to survival, as were primary tumour size ($P=0.0003$), nodal involvement ($P=0.05$) and progesterone receptor status ($P=0.003$). Tumours with grade I and diploid DNA content had a 100% 8-year survival rate (Table II).

### Discussion

The 8-year survival rate was significantly better in breast cancer patients with DNA-diploid as compared to DNA-aneuploid tumours. This is in accordance with previous reports on the relation of DNA ploidy with the relapse-free interval (Hedley *et al.*, 1983; Cornelisse *et al.*, 1987; Dowle *et al.*, 1987; Kallioniemi *et al.*, 1987*a*) and overall survival (Coulson *et al.*, 1984; Cornelisse *et al.*, 1987; Dowle *et al.*, 1987) in breast cancer. However, in one study (Klintenberg *et al.*, 1986) no survival difference between DNA-aneuploid and DNA-diploid breast cancers was observed. Contradictory results regarding the prognostic value of DNA ploidy analysis may be due to differences in the type of treatment given to the patients as well as due to methodological differences causing variation in the proportion of aneuploid cases detected. Long-term follow-up studies with microspectrophotometric DNA measurements (Atkin, 1972; Auer *et al.*, 1980; Auer *et al.*, 1984; Fallenius, 1986) support the prognostic value of DNA ploidy.

Tumours with a hypertetraploid DNA content (DNA index >2.20) were related to a worse, and those with a tetraploid DNA content to a better prognosis, than other DNA-aneuploid tumours. Similar results have been reported by static cytophotometry in breast cancer (Auer *et al.*, 1980; Auer *et al.*, 1984; Fallenius, 1986). Our flow cytometric results in ovarian cancer (Kallioniemi *et al.*, 1987*b*) also indicate that hypertetraploid tumours are highly malignant. In the statistical analyses we treated the DNA-aneuploid tumours as a single group because only 20 tumours contained a hypertetraploid cell clone. A multiploid DNA abnormality did not indicate a worse prognosis than single DNA aneuploidy, which is consistent with the results of Cornelisse *et al.* (1987).

DNA ploidy was not significantly related to menopausal status, which is in accordance with most previous publications (Raber *et al.*, 1982; Ewers *et al.*, 1984; Hedley *et al.*, 1984; Kute *et al.*, 1985). A few investigators have reported a slightly higher occurrence of DNA-aneuploid

**Table III** Univariate and multivariate (Cox model) survival analysis in 297 Stage I–III breast cancer patients. Relative risk of death, its 95% confidence interval and *P* value are given for each covariate

| | *Univariate analyses* | | *Multivariate analyses* | |
|---|---|---|---|---|
| *Variable* | *Relative risk of death* | *P value* | *Relative risk of death* | *P value* |
| *Independently associated with survival:* | | | | |
| DNA ploidy: | | | | |
| Diploid | 1.0 | | 1.0 | |
| Aneuploid | 3.0 (1.8, 5.0) | <0.0001 | 2.2 (1.3, 3.8) | 0.001 |
| Primary tumour size: | | | | |
| T1 | 1.0 | | 1.0 | |
| T2 | 1.7 (0.9, 3.1) | <0.0001 | 1.7 (0.9, 3.1) | 0.0007 |
| T3–4 | 4.3 (2.3, 7.9) | | 3.4 (1.7, 6.7) | |
| Nodal status: | | | | |
| N0 | 1.0 | | 1.0 | |
| N1 | 2.4 (1.6, 3.7) | <0.0001 | 1.9 (1.2, 3.0) | 0.04 |
| N2–3 | 3.7 (1.9, 7.4) | | 1.8 (0.7, 4.2) | |
| Progesterone receptor: | | | | |
| >100 | 1.0 | | 1.0 | |
| 5–100 | 1.7 (1.1, 2.5) | <0.0001 | 1.7 (1.0, 2.5) | 0.0008 |
| <5 | 3.3 (1.7, 5.0) | | 2.5 (1.4, 5.0) | |
| *Associated with survival only when analysed alone:* | | | | |
| Oestrogen receptor: | | | | |
| >100 | 1.0 | | | |
| 5–100 | 1.7 (1.1, 2.5) | 0.02 | | 0.5 |
| <5 | 2.0 (1.1, 3.3) | | | (NS) |
| *Not significantly associated with survival:* | | | | |
| Age: | | | | |
| <50 | 1.0 | | | |
| 50–65 | 1.4 (0.8, 2.2) | 0.2 | | 0.1 |
| >65 | 1.3 (0.8, 2.2) | (NS) | | (NS) |
| Menopausal status: | | | | |
| Premenop. | 1.0 | | | |
| Postmenop. | 1.3 (0.9, 2.0) | 0.2 (NS) | | 0.2 (NS) |

tumours in postmenopausal patients (Thorud *et al.*, 1986; Dowle *et al.*, 1987). Our results indicated that DNA-aneuploid tumours were most common in patients aged 50–65. DNA aneuploidy was significantly more common in node-positive than in node-negative tumours and most common in tumours with extensive (N2–N3) nodal involvement, whereas there was no significant correlation between DNA aneuploidy and primary tumour size. Previous literature on the relation of DNA ploidy to TNM classification is controversial. It has been reported that DNA aneuploidy is related to nodal involvement only (Hedley *et al.*, 1984), to primary tumour size only (Ewers *et al.*, 1984; Fallenius *et al.*, 1986; Thorud *et al.*, 1986; Dowle *et al.*, 1987), to both parameters (Cornelisse *et al.*, 1987) or to neither of them (Taylor *et al.*, 1983; McDivitt *et al.*, 1986; Jakobsen *et al.*, 1984).

The present as well as several previous studies clearly document that poorly differentiated tumours are more often aneuploid than moderately or well-differentiated ones (Olszewski *et al.*, 1981; Moran *et al.*, 1984; Jakobsen *et al.*, 1984; McDivitt *et al.*, 1986; Thorud *et al.*, 1986; Dowle *et al.*, 1987; Kallioniemi *et al.*, 1987*a*). The present study and many (Olszewski *et al.*, 1981; Bichel *et al.*, 1982; Moran *et al.*, 1984; Coulson *et al.*, 1984; Jakobsen *et al.*, 1984; Horsfall *et al.*, 1986; Cornelisse *et al.*, 1987; Kallioniemi *et al.*, 1987*a*) but not all other studies (Raber *et al.*, 1982; Taylor *et al.*, 1983; Hedley *et al.*, 1984; McDivitt *et al.*, 1986; Klintenberg *et al.*, 1986) suggest that DNA aneuploidy is more common in oestrogen receptor negative than in receptor positive tumours. We also observed a highly significant association between DNA aneuploidy and absence of PR receptors, confirming some previous studies (Moran *et al.*, 1984; Coulson *et al.*, 1984; Horsfall *et al.*, 1986; Kallioniemi *et al.*, 1987*a*) yet again disagreeing with some others (Taylor *et al.*, 1983; Jakobsen *et al.*, 1984; McDivitt *et al.*, 1986). Because many of the early studies were based on relatively few patients, a significant association between DNA ploidy and the clinicopathological parameters was not always achieved despite a trend in that direction.

According to our results DNA ploidy had prognostic significance in both pre- and postmenopausal patients, in node-negative and node-positive patients, as well as in primary tumours of all sizes. The results of Cornelisse *et al.* (1987) indicated a less powerful prognostic effect confined mainly to postmenopausal patients with locally advanced disease. Hedley *et al.* (1984) reported that in stage II breast cancer DNA aneuploidy was related to poor prognosis irrespective of the number of affected lymph-nodes. Our results also indicated that oestrogen and especially progesterone receptor negative tumours were associated with a low survival rate irrespective of the DNA ploidy level, whereas in receptor positive tumours DNA ploidy had significant prognostic influence. This suggests that DNA aneuploidy might help to identify those patients with steroid receptor positive tumours, who are unlikely to benefit from endocrine therapy. Although DNA aneuploidy *per se* does not appear to correlate with responsiveness to endocrine therapy (Stuart-Harris *et al.*, 1985), it has been proposed (Bichel *et al.*, 1982; Horsfall *et al.*, 1986) that DNA-aneuploid steroid receptor positive tumours would have a poor response to endocrine therapy.

A Cox regression analysis showed that DNA aneuploidy, tumour size, lymph node and progesterone receptor status were the only independent prognostic factors in breast cancer. Although the axillary node involvement was in all cases verified by a pathologist, the extent of the involvement was determined primarily by clinical examination using the TNM classification. This may explain why nodal status had rather weak prognostic effect in multivariate analyses as compared to e.g. primary tumour size. However, it is unlikely that this would have affected the prognostic impact of DNA ploidy, since DNA ploidy had more prognostic significance in node-negative than in node-positive cases. The multivariate analyses clearly showed that the content of progesterone receptors was a better prognostic indicator than that of oestrogen receptors. The combined analysis of progesterone receptor content and DNA ploidy seems to give the most accurate measure of the biological malignancy of breast tumours. If histological grade was included in the Cox model, both grade and DNA ploidy showed independent prognostic significance. It has been definitely demonstrated that the histological grade of breast cancer is an important prognostic indicator (Davis *et al.*, 1986; Sharkey, 1982), but its clinical use has been questioned due to the subjectivity and interindividual variability (Stenkvist *et al.*, 1979; Davis *et al.*, 1986; Sharkey, 1982) of visual grading. The analysis of DNA ploidy is more objective than the evaluation of grade. Furthermore, the present results indicate that the two parameters complement each other in the prediction of aggressiveness of breast cancer.

Our own previous results (Kallioniemi *et al.*, 1987*a*) as well as those of Dowle *et al.* (1987) and Cornelisse *et al.* (1987) have shown that differences in the disease-free survival between DNA-diploid and DNA-aneuploid breast tumours are greater than those in overall survival. Dowle and Cornelisse have also reported that differences in survival between the two ploidy groups decreased after 6–7 years follow-up. In the present study we observed a constantly improved survival in patients with DNA-diploid tumours up to 8 years postoperatively. However, the follow-up period should be further extended, because deaths due to breast cancer are noted up to 20 years after initial diagnosis (Sutherland & Mather, 1986; Harris & Hellman, 1986). The association between DNA aneuploidy and high *S*-phase fraction (Kallioniemi *et al.*, 1987*a*) could explain why the survival difference between DNA-diploid and DNA-aneuploid tumours is most prominent after short-term follow-up.

In conclusion, our results indicate that DNA ploidy is an independent prognostic factor in breast cancer. The combination of DNA ploidy and the content of progesterone receptors offers the best estimate of the biological malignancy of cancer cells, which supplements the information obtained from the TNM classification as well as the histological grading. It remains to be determined how DNA ploidy is related to the responsiveness of a steroid receptor positive breast cancer to endocrine therapy and what is the prognostic value of DNA ploidy after very long follow-up times.

We would like to thank Professor Timo Hakulinen for advice on the statistical analyses of survival data, Mrs Leena Pankko and Ms Eeva Särkkä for excellent technical assistance. This work was supported by grants from the Tampere Research Foundation, The Pirkanmaa Foundation of the Finnish Cancer Society and the Medica Research Foundation.

### References

ATKIN, N.B. (1972). Modal deoxyribonucleic acid value and survival in carcinoma of the breast. *Br. Med. J.*, **1**, 271.

AUER, G.U., CASPERSSON, T.O. & WALLGREN, A.S. (1980). DNA content and survival in mammary carcinoma. *Anal. Quant. Cytol.*, **2**, 161.

AUER, G., ERIKSSON, E., AZAVEDO, E., CASPERSSON, T. & WALLGREN, A. (1984). Prognostic significance of nuclear DNA content in mammary adenocarcinomas in humans. *Cancer Res.*, **44**, 394.

BICHEL, P., POULSEN, H.S. & ANDERSEN, J. (1982). Estrogen receptor content and ploidy of human mammary carcinoma. *Cancer*, **50**, 1771.

BLANCO, G., ALAVAIKKO, M., OJALA, A. & 5 others (1984). Estrogen and progesterone receptors in breast cancer: Relationships to tumour histopathology and survival of patients. *Anticancer Res.*, **4**, 383.

CORNELISSE, C.J., VAN DE VELDE, C.J.H., CASPERS, R.J.C., MOOLENAAR, A.J. & HERMANS, J. (1987). DNA ploidy and survival in breast cancer patients. *Cytometry*, **8**, 225.

COULSON, P.B., THORNTHWAITE, J.T., WOOLLEY, T.W., SUGARBAKER, E.V. & SECKINGER, D. (1984). Prognostic indicators including DNA histogram type, receptor content, and staging related to human breast cancer survival. *Cancer Res.*, **44**, 4187.

COX, D.R. (1972). Regression models and life-tables. *J. R. Stat. Soc.* (*B*), **34**, 187.

DAVIS, B.W., GELBER, R.D., GOLDHIRSCH, A. & 8 others (1986). Prognostic significance of tumor grade in clinical trials of adjuvant therapy for breast cancer with axillary lymph node metastasis. *Cancer*, **58**, 2662.

DIXON, W.J. (1983). *BMDP Statistical Software*. Berkely, Los Angeles, London: University of California Press.

DOWLE, C.S., OWAINATI, A., ROBINS, A. & 4 others (1987). Prognostic significance of the DNA content of human breast cancer. *Br. J. Surg.*, **74**, 133.

EWERS, S.-B., LÅNGSTRÖM, E., BALDETORP, B. & KILLANDER, D. (1984). Flow-cytometric DNA analysis in primary breast carcinomas and clinicopathological correlations. *Cytometry*, **5**, 408.

FALLENIUS, A. (1986). *DNA Content and Prognosis in Breast Cancer* (thesis). Stockholm.

HARRIS, J.R. & HELLMAN, S. (1986). Observations on survival curve analysis with particular reference to breast cancer treatment. *Cancer*, **57**, 925.

HEDLEY, D.W., FRIEDLANDER, M.L., TAYLOR, I.W., RUGG, C.A. & MUSGROVE, E.A. (1983). Method for analysis of cellular DNA content of paraffin-embedded pathological material using flow cytometry. *J. Histochem. Cytochem.*, **31**, 1333.

HEDLEY, D.W., RUGG, C.A., NG, A.B.P. & TAYLOR, I.W. (1984). Influence of cellular DNA content on disease-free survival of stage II breast cancer patients. *Cancer Res.*, **44**, 5395.

HORSFALL, D.J., TILLEY, W.D., ORELL, S.R., MARSHALL, V.R. & McK. CANT, E.L. (1986). Relationship between ploidy and steroid hormone receptors in primary invasive breast cancer. *Br. J. Cancer*, **53**, 23.

JAKOBSEN, A., POULSEN, H.S., MADSEN, E.L., PETERSEN, S.E. & HANSEN, H.S. (1984). Ploidy level of human breast carcinoma. Relation to histopathologic features and hormone receptor content. *Acta Radiol. Oncol.*, **23**, 103.

KALLIONIEMI, O.-P., HIETANEN, T., MATTILA, J., LEHTINEN, M., LAUSLAHTI, K. & KOIVULA, T. (1987*a*). Aneuploid DNA content and high S-phase fraction of tumour cells are related to poor prognosis in patients with primary breast cancer. *Eur. J. Cancer Clin. Oncol.*, **23**, 277.

KALLIONIEMI, O.-P., PUNNONEN, R., MATTILA, J., LEHTINEN, M. & KOIVULA, T. (1987*b*). Prognostic significance of DNA index, multiploidy and S-phase fraction in ovarian cancer. *Cancer*, (in press).

KLINTENBERG, C., STÅL, O., NORDENSKJÖLD, B., WALLGREN, A., ARVIDSSON, S. & SKOOG, L. (1986). Proliferative index, cytosol estrogen receptor and axillary node status as prognostic predictors in human mammary carcinoma. *Breast Cancer Res. Treat.*, **7** (Suppl.), 99.

KUTE, T.E., MUSS, H.B., HOPKINS, M., MARSHALL, R., CASE, D. & KAMMIRE, L. (1985). Relationship of flow cytometry results to clinical and steroid receptor status in human breast cancer. *Breast Cancer Res. Treat.*, **6**, 113.

McDIVITT, R.W., STONE, K.R., CRAIG, B., PALMER, J.O., MEYER, J.S. & BAUER, W.C. (1986). A proposed classification of breast cancer based on kinetic information. *Cancer*, **57**, 269.

McGUIRE, W.L. & DRESSLER, L.G. (1985). Emerging impact of flow cytometry in predicting recurrence and survival in breast cancer patients. *J. Natl Cancer Inst.*, **75**, 405.

MORAN, R.E., BLACK, M.M., ALPERT, L. & STRAUS, M.J. (1984). Correlation of cell-cycle kinetics, hormone receptors, histopathology, and nodal status in human breast cancer. *Cancer*, **54**, 1586.

OLSZEWSKI, W., DARZYNKIEWICZ, Z., ROSEN, P.P., SCHWARTZ, M.K. & MELAMED, M.R. (1981). Flow cytometry of breast carcinoma: I. Relation of DNA ploidy level to histology and estrogen receptor, *Cancer*, **48**, 980.

RABER, M.N., BARLOGIE, B., LATREILLE, J., BEDROSSIAN, C., FRITSCHE, H. & BLUMENSCHEIN, G. (1982). Ploidy, proliferative activity and estrogen receptor content in human breast cancer. *Cytometry*, **3**, 36.

SCARFF, R.W. & TORLONI, H. (1968). *Histological Typing of Breast Tumours*. International Classification of Tumours No. 2. Histological typing of breast tumours. WHO, Geneva.

SHARKEY, F.E. (1982). Biological meaning of stage and grade in human breast cancer: Review and hypothesis. *Breast Cancer Res. Treat.*, **2**, 299.

STENKVIST, B., WESTMAN-NAESER, S., VEGELIUS, J. & 4 others (1979). Analysis of reproducibility of subjective grading systems for breast carcinoma. *J. Clin. Pathol.*, **32**, 979.

STUART-HARRIS, R., HEDLEY, D.W., TAYLOR, I.W., LEVENE, A.L. & SMITH, I.E. (1985). Tumour ploidy, response and survival in patients receiving endocrine therapy for advanced breast cancer. *Br. J. Cancer*, **51**, 573.

SUTHERLAND, C.M. & MATHER, F.J. (1986). Long-term survival and prognostic factors in breast cancer patients with localized (no skin, muscle, or chest wall attachment) disease with and without positive lymph nodes. *Cancer*, **57**, 622.

TAYLOR, I.W., MUSGROVE, E.A., FRIEDLANDER, M.L., FOO, M.S. & HEDLEY, D.W. (1983). The influence of age on the DNA ploidy levels of breast tumours. *Eur. J. Cancer Clin. Oncol.*, **19**, 623.

THORUD, E., FOSSÅ, S.D., VAAGE, S. & 4 others (1986). Primary breast cancer. Flow cytometric DNA pattern in relation to clinical and histopathologic characteristics. *Cancer*, **57**, 808.

VIHKO, R., JÄNNE, O., KONTULA, K. & SYRJÄLÄ, P. (1980). Female sex steroid receptor status in primary and metastatic breast carcinoma and its relationship to serum steroid and peptide hormone levels. *Int. J. Cancer*, **26**, 13.